# Stream water age distributions controlled by storage dynamics and nonlinear hydrologic connectivity: Modeling with high‐resolution isotope data

**DOI:** 10.1002/2015WR017888

**Published:** 2015-09-26

**Authors:** C. Soulsby, C. Birkel, J. Geris, J. Dick, C. Tunaley, D. Tetzlaff

**Affiliations:** ^1^ Northern Rivers Institute, School of Geosciences, University of AberdeenUK; ^2^ Department of GeographyUniversity of Costa Rica, San Pedro Montes de OcaCosta Rica

**Keywords:** water age, rainfall‐runoff model, tracers, stable isotopes, connectivity

## Abstract

To assess the influence of storage dynamics and nonlinearities in hydrological connectivity on time‐variant stream water ages, we used a new long‐term record of daily isotope measurements in precipitation and streamflow to calibrate and test a parsimonious tracer‐aided runoff model. This can track tracers and the ages of water fluxes through and between conceptual stores in steeper hillslopes, dynamically saturated riparian peatlands, and deeper groundwater; these represent the main landscape units involved in runoff generation. Storage volumes are largest in groundwater and on the hillslopes, though most dynamic mixing occurs in the smaller stores in riparian peat. Both streamflow and isotope variations are generally well captured by the model, and the simulated storage and tracer dynamics in the main landscape units are consistent with independent measurements. The model predicts that the average age of stream water is ∼1.8 years. On a daily basis, this varies between ∼1 month in storm events, when younger waters draining the hillslope and riparian peatland dominates, to around 4 years in dry periods when groundwater sustains flow. This variability reflects the integration of differently aged water fluxes from the main landscape units and their mixing in riparian wetlands. The connectivity between these spatial units varies in a nonlinear way with storage that depends upon precipitation characteristics and antecedent conditions. This, in turn, determines the spatial distribution of flow paths and the integration of their contrasting nonstationary ages. This approach is well suited for constraining process‐based modeling in a range of northern temperate and boreal environments.

## Key Points

- Stream water age is estimated by modeling high‐resolution isotope time series
- Storage dynamics drive nonlinear spatial patterns of hydrologic connectivity
- Connectivity variations determine nonstationary stream water ages

## 1 Introduction

Using tracers in conceptual models to explore the relationships between water and solute travel times has a long history [e.g., see *Neal et al*., 1988; *Barnes and Bonell*, 1996; *Weiler et al*., 2003, and review by *Birkel and Soulsby*, 2015]. More recently, a number of applications have focused on using tracer‐aided models to estimate time variance in stream water age distributions through tracking water and conservative tracer fluxes through conceptual storages and assessing the associated mixing processes [*Birkel et al*., 2011a, 2011b, 2014, 2015; *McMillan et al*., 2012; *Davies et al*., 2013; *Hrachowitz et al*., 2013; *Benettin et al*., 2015]. Such coupled flow‐tracer models are useful process‐based complementary approaches to recent advances in more analytical methods for understanding the relationships between catchment storage dynamics, mixing, and transit times [*Botter et al*., 2010; *Botter*, 2012; *Harman*, 2015]. A key aspect in both approaches is how the modeling captures nonlinearities and nonstationarities in catchment storage dynamics and hydrological response and conceptualizes the associated tracer flux to streams [e.g., *Heidbuechel et al*., 2012].

Progress in stable isotope analytical methods means that the availability and quality of tracer data is often now more closely suited to the temporal capabilities of rainfall‐runoff modeling. Isotope measurements for daily and subdaily precipitation and streamflow samples (rather than weekly) is becoming more readily available for multiyear periods at a number of research sites [*Kirchner and Neal*, 2013; *Pangle et al*., 2013; *Birkel et al*., 2014]. Integrating such tracer data in rainfall‐runoff models provided a new opportunity for enhancing these models as learning frameworks for hypothesis testing on the processes governing water and solute fluxes; as well as identifying weaknesses with models conditioned on more coarsely sampled data [*McGuire et al*., 2007]. While much progress has been made in modeling input‐output relationships and inferring processes, little of this has been spatially explicit [*Birkel and Soulsby*, 2015]. A research priority is conceptualizing the heterogeneity in catchment landscapes, the nonlinear connectivity between different landscape units and how this interacts to govern the spatial and temporal variability of hydrological response and tracer transport [e.g., *Jencso et al*., 2010; *Tetzlaff et al*., 2014]. While empirical studies have provided a physical basis for such conceptualization, there has been less progress in implementing this in a tracer‐aided modeling framework. Traditional lumped approaches have usually looked at catchment scales [e.g., *McMillan et al*., 2012; *Hrachowitz et al*., 2013], even though it has been shown that the internal function of catchments results in marked spatial and temporal variability in how landscape units partition, store, and release water [e.g., *Iorgulescu et al*., 2007; *Dunn et al*., 2010] which affects the scaling of advection‐dispersion processes [*Kirchner et al*., 2001]. Such interactions are usually nonlinear and have major implications for nonstationarity in mixing and transport processes as well as time variance in transit times [e.g., *Soulsby et al*., 2015]. Characterizing such interactions in a spatially explicit way, but with minimal parameterization, remains a major challenge in water and solute modeling [*Wissmeier and Uhlenbrook*, 2007].

Here we report advances in integrating new field insights in models, motivated by new, high‐resolution tracer data from a wet, peatland‐dominated experimental site in Scotland. Previous work has related nonlinear runoff responses to the connectivity of different hydrological response units [*Tetzlaff et al*., 2007, 2014; *Ali et al*., 2014]. In the past, we have used conceptual models to explore the routing of conservative tracers in terms of the geographic source areas of runoff [*Tetzlaff et al*., 2008; *Birkel et al*., 2010] and their associated travel times [*Birkel et al*., 2011a, 2011b]. The internal consistency of these model structures and identifiability of parameters have also been tested against measured catchment states in terms of water table levels and soil water isotopes [*Birkel et al*., 2014]. We have also used these models to explore the general relationships between nonstationarity in tracer fluxes and catchment transit times during wetter and drier years over a long (6 year) period of record of tracer data [*Birkel et al*., 2015]; however, this work was limited by a coarse (weekly) sampling resolution. Despite such limitations, the models have been sufficiently robust to provide a hydrological framework for simple biogeochemical models that have been successfully used in water quality simulations for dissolved organic carbon (DOC) [*Birkel et al*., 2014; *Dick et al*., 2015].

Here we test the modeling approach with a new, unique multiyear data set of daily tracer measurements in precipitation and streamflow. These data have been collected in a small (3.2 km^2^) catchment where extensive tracer data are also available for soil waters and groundwaters, as well as soil moisture content and groundwater level data. Integrating these new data with the model allows us, for the first time, to examine the interaction between nonstationary catchment transit times, water age distributions of different internal fluxes, and nonlinearity in storage‐driven hydrological connectivity. The aim of the paper is to use a coupled flow and tracer model to address the following questions:

1. How well can we simulate daily runoff and tracer dynamics using a parsimonious model in a way that is consistent with measurements of state variables?
2. How do the ages of water fluxes from different conceptual stores (i.e., landscape units) vary and then integrate at the catchment scale to control stream water age?
3. How does this integration vary under different hydroclimatic conditions and what are the associated storage dynamics that control nonlinearities in hydrological connectivity?

From this, we also discuss the challenges and prospects for using conceptual models in transit time modeling. While the work in hand is specific to a small catchment in the Scottish Highlands, the approach is intended to be particularly relevant to northern temperate and boreal landscapes where peaty soils contribute to runoff generation and generic applicability wherever dynamic saturation zones are important.

## 2 Study Site

**Figure 1:**
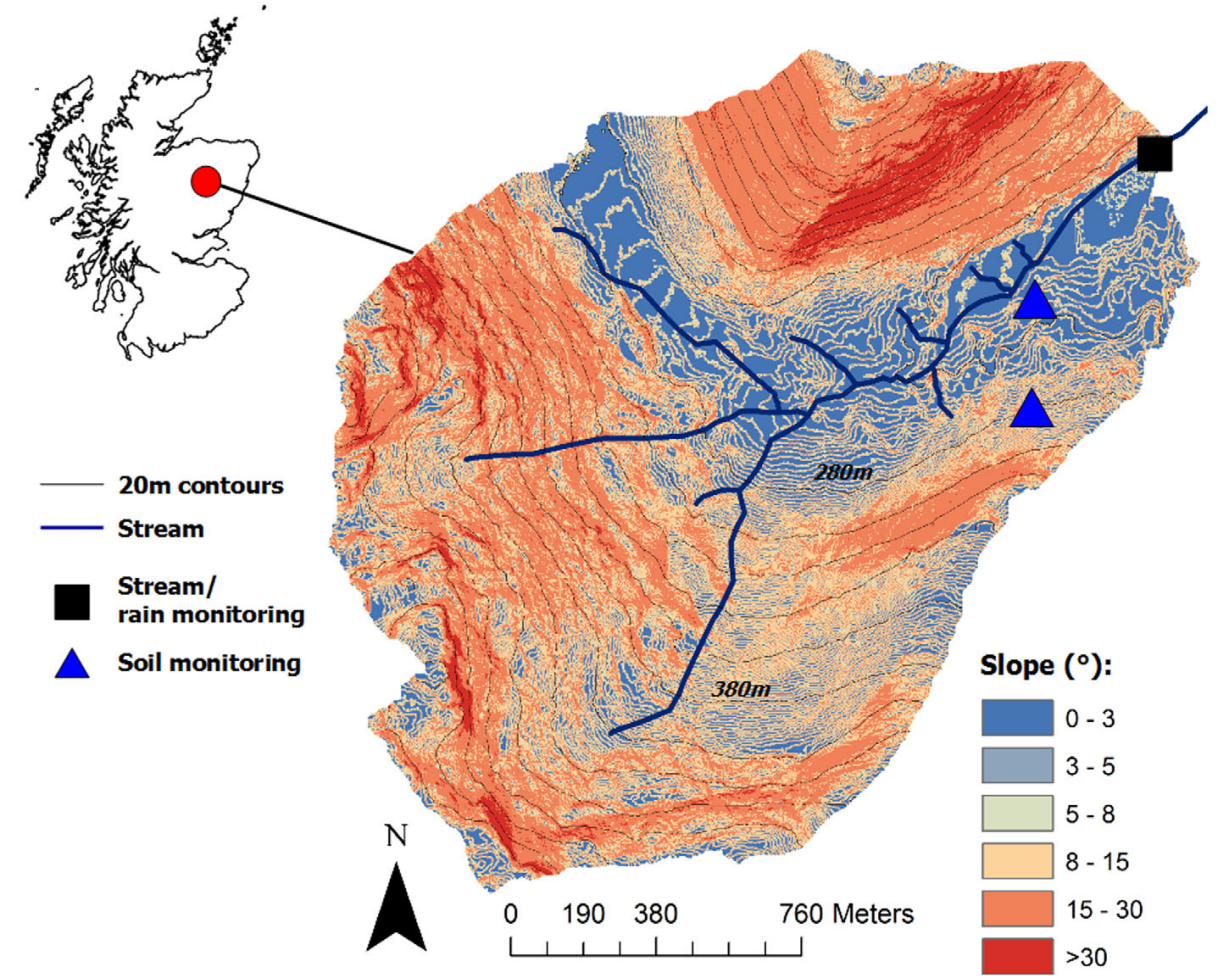
Location, topography, and monitoring network of the Bruntland Burn experimental catchment in Scotland. The microscale slope classification was calculated from a 1 m^2^ LiDAR‐derived digital elevation model indicating the main landscape units of hillslope and valley bottom.

The Bruntland Burn is a 3.2 km^2^ montane catchment (elevation range 240–580 m) in the Scottish Highlands (Figure 1). It is described in detail elsewhere [e.g., *Birkel et al*., 2011a; *Tetzlaff et al*., 2014; *Blumstock et al*., 2015]. Briefly, the climate is temperate/boreal oceanic; mean annual temperatures are 6°C, varying between daily means of 1°C and 12°C in January and July, respectively. Periods of temperatures continually below 0°C are usually limited to a week or less, and day‐time temperatures rarely drop below −5°C, so periods of soil frost are transient and mostly only affect elevations above 450 m [*Hannah et al*., 2008]. Mean annual precipitation is around 1000 mm; usually <5% falls as snow, though this can be as high as 10%. Most precipitation falls as low intensity rain (approximately 50% of the annual total falls in events of <10 mm d^−1^), which is fairly evenly distributed throughout the year, though the period November–February usually has the highest precipitation and runoff events. Annual potential evapotranspiration is around 400 mm and annual runoff is around 700 mm. Runoff event coefficients vary between around 10% with dry antecedent conditions (usually in summer) and ∼40% in wetter periods.

The solid geology is characterized by low permeability, with granite to the north and metamorphic rocks to the south. The stream occupies a glaciated valley with steep slopes and a flat valley bottom. Around 70% of the catchment is covered by glacial drift deposits; mainly undifferentiated till which comprises pebble to boulder sized clasts in a silty‐sand matrix. The depth ranges from around 5 m on the steeper hillslopes, to around 40 m in the valley bottom. The till is the main aquifer for groundwater storage and has a porosity of around 20%, but low permeability with tentative hydraulic conductivity estimates ranging from 10^−6^ to 10^−4^ m s^−1^ [*Malcolm et al*., 2004]. Soil cover reflects topography: on steep slopes (>8°) freely draining podzols (∼0.7 m deep) or rankers (<0.3 m deep) dominate; the flat (<3°) valley bottom is characterized by deep (>1 m deep) peat soils. The peat thins to around 0.3 m on the intermediate slopes, with peaty gley soils dominating [*Tetzlaff et al*., 2007]. The peats have a massive soil structure and limited vertical drainage, with most water movement being lateral in the upper 0.2 m. In contrast, the podzols are characterized by vertical drainage. The vegetation reflects soil characteristics with *Spagnum* spp. mosses and grasses like *Molinia caerulea* dominating the peatland communities with a canopy ∼0.2 m high and heather (*Calluna vulgaris* and *Erica tetralix*) scrubs ∼0.3–0.4 m high dominating the podzols. The heather roots create a moderate amount macroporosity in the upper 0.3 m of the profile. This mainly facilitates vertical drainage, but in the wettest conditions (when the soil is saturated) can cause lateral flow downslope toward the riparian peatland. Trees, mainly Scots pine (*Pinus sylvestris*) cover about 10% of the catchment mainly on steeper slopes or on plantations.

Previous work has shown that the hydrological response of the Bruntland Burn is strongly dependent on the connectivity between the steeper podzols and peats in the riparian wetland [*Birkel et al*., 2014]. The extent of saturation in the riparian wetland expands and contracts in response to antecedent wetness and event precipitation [similar to *Dunne et al*., 1975]. In wet periods, the saturation zone was observed to extend to 40% of the catchment area and the connectivity between the hillslopes and wetland is strong as a result of lateral flow [*Ali et al*., 2014]. This drives the main storm period response of the catchment, with runoff being generated by saturation overland flow from this expanding saturation zone which is fed by seepage from upslope [*Tetzlaff et al*., 2014]. In drier periods, the saturation zone can be as low as 2% of the catchment and the hillslopes can become disconnected. At these times, streamflow is sustained mainly from groundwater in the drift [*Soulsby et al*., 1998; *Blumstock et al*., 2015]. However, precipitation events following dry antecedent periods mainly generate runoff from the saturated area. At such times, precipitation on the drier hillslopes simply replenishes soil moisture deficits [*Tetzlaff et al*., 2014; *Geris et al*., 2015].

Previous work has shown that isotopes in precipitation have a distinct seasonal pattern with δ^2^H ranging from ∼−150‰ in winter to ∼−10‰ in summer [*Tetzlaff et al*., 2014]. This variability is greatly damped in stream water which only varies between around ∼−70‰ to ∼−50‰; most of this range reflects seasonal variability, and event‐scale changes are usually modest [*Birkel et al*., 2011a]. This has been explained by the high water storage in the riparian peatlands and the mixing and damping of variable isotope inputs which hydrograph separations have shown to result in “old” or “preevent” water dominating (usually >90%) the storm hydrograph [*Tetzlaff et al*., 2014].

## 3 Data and Modeling Approach

### 3.1 Data

The hydrometric and isotope data available for the Bruntland Burn are as follows (and see Table 1). Daily precipitation amounts were estimated over 11 hydrological years from 1 October 2003 to 30 September 2014. Meteorological data are mainly based on a Campbell automatic weather station (AWS) 1 km away that is operated by Marine Science Scotland (MSS) as well as snow depth data at Balmoral, some 5 km away (British Atmospheric Data Centre, BADC). The data from the AWS were used to calculate potential evapotranspiration (PET) using the Penman‐Monteith equation. Daily average temperature was altitudinally corrected and precipitation interpolated using five surrounding rain gauges (Scottish Environmental Protection Agency, SEPA). The interpolation was performed using an inverse distance elevation gradient algorithm similar to *Capell et al*. [2012]. Discharge measurements commenced in October 2007 and were derived from 15 min water level records (using an Odyssey capacitance probe) in a stable, rated section. Groundwater levels (Odyssey capacitance probes) and soil moisture (Campbell time domain reflectometers) measurements (15 min intervals) were also collected at the sites along the hillslope transect from June 2011 and collection continues to the present [*Geris et al*., 2015].

**Table 1:** Summary Statistics (Mean and Standard Deviation) of Daily Data Used for This Analysis Separated Into Calibration (1 June 2011 to 30 September 2014) and Test (1 October 2007 to 30 September 2009) Periods

	dSAT	Q	P	PET	SWE	T	P_D	Q_D	Hist@10cm	Podzol@10cm	SM@Hist	SM@Podzol
Unit		mm/d	mm/d	mm/d	mm/d	°C	‰	‰	‰	‰	v/v	v/v
Calibration	0.13 (0.11)	1.9 (2.1)	3 (5.4)	1.4 (1.2)	0.07 (0.8)	7.4 (5)	−51.6 (19.4)	−57.2 (3.1)	−54.6 (4.1)	−55.5 (9.5)	0.82 (0.02)	0.33 (0.03)
Test period	0.11 (0.08)	1.7 (1.4)	2.4 (4.6)	1 (0.9)	0.14 (1.2)	6.6 (5.3)	−50.6 (18.5)	−57 (2.8)				

For stable isotopes, weekly precipitation sampling started at the Bruntland Burn in October 2003. However, from October 2007, precipitation samples have been collected daily. Occasional gaps in the precipitation record reflect occurrences of sampler failure or site inaccessibility in bad weather. Stream water samples were collected on a weekly basis from October 2007, and then daily between October 2008 and the end of 2009 using an ISCO 3700 automatic water sample. After an 18 month hiatus (due to a funding gap), daily stream sampling restarted on 1 June 2011 and has continued since. Additionally, weekly or fortnightly soil isotope (using Rhizon soil samplers) sampling in three soil profiles (10, 30, and 50 cm depth each) of a representative hillslope transect (encompassing the catena transition from podzols to peat) started in June 2011 and continued until October 2013 [*Tetzlaff et al*., 2014]. Weekly groundwater samples were also collected from two wells draining at least 5 m of drift for most of this period. All stable isotope samples were analyzed with a Los Gatos DLT‐100 laser isotope analyzer (precision ±0.1‰ for oxygen‐18 (δ^18^O); ±0.4 ‰ for deuterium (δ^2^H)). Isotope signatures are reported in the δ‐notion (‰) after calibration using Vienna Standard Mean Ocean Water (VSMOW) standards. Because of the higher relative precision, we only used δ^2^H in the modeling though δ^18^O had a similar variation.

To complete the precipitation daily isotope time series, a multiple linear regression (MLR) gap filling procedure was developed and used, though this accounted for <5% of the time series. MLR models based on meteorological variables (temperature, humidity, solar radiation, and precipitation volume) were constructed to simulate δ^2^H and δ^18^O in precipitation. The best fit (adjR^2^ = 0.47) and most parsimonious model used air temperature and relative humidity as explanatory variables. This procedure mimics the observed natural variability in precipitation isotopes and avoids the previously applied gap filling using the same isotopic value of the neighboring measurement.

### 3.2 Modeling Approach

The model presented here was developed in previous work in the Bruntland Burn catchment that has used tracer data and other empirical measurements in addition to stream discharge to constrain model structures, improve parameterization, and aid calibration [*Birkel et al*., 2011a, 2011b, 2014, 2015]. The reader is referred to these original papers for full details. These models were initially developed to simulate streamflow and stream alkalinity based on measured source water composition [*Birkel et al*., 2010]. Stable isotopes in streamwaters were subsequently added to simulate the temporal dynamics of the transfers of water and tracers in the rainfall‐runoff transformation and the mixing processes involved [*Birkel et al*., 2011a, 2011b]. Further model developments included integration of soil isotope data, soil moisture, and groundwater levels to test model structures, calibration objectives, and parameter identifiability [*Birkel et al*., 2014]. Here we were able to use the long‐term daily isotope time series to drive a much more constrained modeling process.

**Figure 2:**
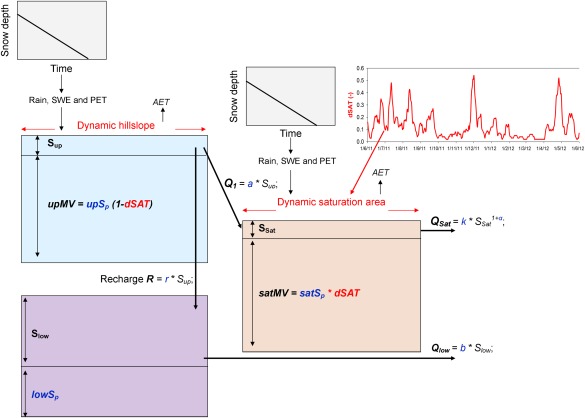
The landscape‐based dynamic model concept is visualized in form of its storage components (hillslope, groundwater, and saturation area) and the simple snow depth‐based snowmelt routine. The dynamic model structure is based on the daily estimated catchment saturation area extent (dSAT), which is used directly as model input. The five calibrated rainfall‐runoff parameters (*a*, *r*, *b*, *k*, and *α*) are shown in blue with the respective water flux equations. The additional mixing volume parameters (*upS_p_*, *lowS_p_*, and *satS_p_*) are given inside the respective storage units indicating the coupled tracer transport routine. SWE is snow water equivalent, AET and PET are actual and potential evapotranspiration, respectively.

Central to all these models was capturing the nonlinear streamflow response by successfully conceptualizing the hydrological connectivity of the catchment which links the hillslopes (Up) to the riparian zone (Sat) and underlying groundwater (Low) (Figure 2). This connects two upper storage units which conceptualize the riparian peat soils and the freely draining podzols on the hillslopes. Direct mapping of the spatial extent of saturated soils in the valley bottom that were hydrologically connected to the stream network during different wetness conditions [see *Ali et al*., 2014] allowed us to develop and fit a simple antecedent precipitation index‐type algorithm [*Birkel et al*., 2010]. This algorithm was applied to create a continuous time series of the expanding and contracting daily saturation area extent (dSAT) (Figure 2). This dSAT time series was used as model input to dynamically distribute daily precipitation inputs between the storage volumes in the landscape‐based (hillslope (S_up_) and saturation area (S_sat_) model structure. Consequently, catchment precipitation *P* and potential evapotranspiration *PET* were distributed into hillslope (*P_up_*, *ET_up_*) and saturation area (*P_sat_*, *ET_sat_*) according to the extent of *dSAT* (only the equations for precipitation are given for illustration here):

(1) $$P_{up}=P(1-dSAT)$$

(2) $$P_{sat}=1-P_{up}$$

Similar to *Birkel et al*. [2015], we used reservoirs that could become unsaturated allowing storage deficits to develop. The riparian area was normally saturated but could have small deficits following prolonged dry periods in summer. In the upper stores, water levels below a certain threshold can only be further depleted by transpiration and no lateral flow will be generated. Incoming precipitation fluxes are first intercepted and reduced by PET if available. The remaining water fills the uppermost storages and reflects soil moisture‐related threshold processes of runoff generation [*Tetzlaff et al*., 2014]. Consequently, S_up_ was often in deficit, but in wetter periods would fill and spill into S_sat_, which usually has low or no deficit and usually generated streamflow. The storages *S* are model state variables and we describe the following fluxes and calibrated parameters. The unsaturated hillslope reservoir *S_up_* is drained (flux *Q*_1_ in mm d^−1^) by the linear rate parameter *a* (day^−1^) and directly contributes to the saturation area store *S_sat_*; the recharge rate *R* (mm d^−1^) to the groundwater reservoir *S_low_* is linearly calculated using the parameter *r* (day^−1^); the *S_low_* generates runoff *Q_low_* (mm d^−1^) contributing to total streamflow *Q* (mm d^−1^) using the linear rate parameter *b* (day^−1^). The runoff component *Q_sat_* (mm d^−1^) generated nonlinearly from *S_sat_* conceptualizes saturation overland flow using the rate parameter *k* (day^−1^) and the nonlinearity parameter *α* in a power function‐type equation (Figure 2); and *Q* is simply the sum of *Q_sat_* and *Q_low_*. The selection of linear or nonlinear parameters was based on previous stepwise multimodel testing for tracer‐aided modeling for similar catchments in the Scottish Highlands [*Birkel et al*., 2010; *Capell et al*., 2012]. In particular, the nonlinear conceptualization of *Q_sat_* has a physical basis in the dynamic expansion of the saturation area and fluxes that generate storm runoff. Likewise, the linear nature of Q1 and R reflect the physical nature of the threshold‐like response of the hillslope fluxes and the low aquifer drainage.

Thus, the basic rainfall‐runoff model uses only five calibrated parameters (*a*, *b*, *r*, *k*, and *α*) shown in blue in Figure 2. The model is configured such that S*_up_* does not contribute directly to streamflow as the steeper hillslopes in the catchment are separated from the channel network by the riparian zone represented by S*_sat_*. Further S*_sat_* is not conceptualized to drain into S*_low_* as the peat soils are saturated with lower subsoil permeability, limiting vertical drainage, and promoting lateral flow [*Tetzlaff et al*., 2014]. Because the modeling was carried out at daily time steps and the catchment was small (3.2 km^2^), we did not need to include a channel routing parameter.

Although snow does not comprise a significant part of the water balance (<5%), transient snow packs can accumulate, though these typically melt in a week or so, and snowmelt can occasionally generate important stormflow responses in winter and spring. Therefore, we adopted a simple snowmelt routine based on a “master snow depth recession curve” taken from recorded daily snow depth at Balmoral (5 km from the BB) similar to *Dick et al*. [2015]. The resulting recession coefficient of 0.15 was used as a fixed melt rate parameter in the additional snow storage that recharges *S_up_* and *S_sat_*.

Importantly, each of the three reservoirs (*S_up_*, *S_sat_*, and *S_low_* in mm) incorporates an additional calibrated storage parameter (*upS_p_*, *satS_p_*, and *lowS_p_*, respectively, in mm) for isotope transport simulations (also shown in Figure 2 in blue). Previous work showed that this was essential, as well as physically meaningful, to capture the importance of the large storage volumes which were needed to damp tracer outputs relative to inputs even though the dynamic storage changes needed to account for runoff variation was much lower [*Birkel et al*., 2011a, 2011b; *Soulsby et al*., 2011]. This was achieved by creating an additional volume available for isotope storage, mixing, and transport that does not affect the dynamic water storage and fluxes:

(3) $$\frac{d\left(c_{}S_{}\right)}{dt}=\sum_{j}c_{I,j}I_{j}-\sum_{k}c_{O,k}O_{k}$$

with *c* being the δ^2^H signature of storage components (‰) in *j* storage inflows *I_j_* (e.g., *P*, *Q_up_*, and *R*) and *k* outflow *O_k_* components (e.g., *ET*, *Q_low_*, and *Q_sat_*) which characterizes the catchment storage *S* dynamics (sum of dynamic and additional storage available for mixing) and associated isotope signature *c*. Additional storage parameters were subsequently converted into time‐variable mixing volumes (*MV*). This was achieved using the nonlinear expansion and contraction of the saturation zone as a means of relaxing the assumption of complete mixing by varying the contributions from the hillslope and saturation area stores at the catchment scale without introducing new parameters. Thus, as the rainfall‐runoff model dynamically varies the extent of the *S_sat_* and *S_up_*, the complete mixing in individual reservoirs is integrated at the catchment scale in a nonlinear manner. The *MVs*, therefore, represent a partial mixing mechanism and were calculated according to the catchment wetness (*dSAT*) state assuming that greater wetness results in greater saturation area extent but also greater potential for mixing (*satMV*). In turn, it is assumed that the hillslope mixing volume (*upMV*) decreased as the saturation area expands (Figure 2).

(4) $$satMV=satS_{p}dSAT$$

(5) $$upMV=upS_{p}(1-dSAT)$$

This conceptualization of mixing is consistent with our goal of maintaining a minimal number of parameters while still capturing the mixing inherent in the advective‐dispersive processes at the hillslope scale [*Kirchner et al*., 2001; *Tetzlaff et al*., 2014].

Isotope tracers can exhibit nonconservative behavior as a result of fractionation processes. This has been observed during summer in superficial waters of the saturation area giving a significant change of slope for streamflow relative to the Local Evaporation Line [*Birkel et al*., 2011a]. Thus, we incorporated evaporative fractionation processes in *S_up_* and *S_sat_* according to *Gibson and Edwards* [2002] and *Birkel et al*. [2011a]. This allows simulation of preferential enrichment in soil waters particularly during periods of high *PET*.

The age of waters in flux is tracked using a time stamp tagging each daily incoming flux and outflowing flux via their movement through the storage cascade similar to *Hrachowitz et al*. [2013]:

(6) $$p_{F,Q}\left(t_{j}-t_{i}{,}_{}t_{j}\right)=\sum_{n=1}^{N}p_{F,Q_{n}}\left(t_{j}-t_{i}{,}_{}t_{j}\right)\frac{Q_{n}\left(t_{j}\right)}{Q\left(t_{j}\right)}$$

where *p_F_*_,_ *_Q_* is the distribution of water age of all contributing fluxes *Q_n_* to total discharge *Q* with *t_j_* being the time of exit at the catchment outlet and *t_i_* the time of entry with *P*. Water flux ages were calculated and visualized as cumulative distribution functions for comparative purposes.

The model was calibrated (parameters *a*, *b*, *r*, *k*, *α*, *upS_p_*, *satS_p_*, and *lowS_p_*) using a multiobjective (considering discharge and tracer) nondominated sorting genetic algorithm (NSGA2) for optimization [*Deb et al*., 2002], applied to the period from 1 June 2011 until 30 September 2014. The calibration procedure based on the recommendations by *Deb et al*. [2002] and included 500 parameter sets defined as a population. The first population was then constrained over 100 iterations, which resulted in a total of 50,000 tested different parameter sets. The final and 500 best parameter combinations which showed strong convergence were retained to calculate posterior parameter variability, to simulate model output and to test against other data periods (test period from 1 October 2007 until 30 September 2009). In the absence of a formal uncertainty analysis, this procedure was assumed to generate a representative subset of parameter combinations able to adequately represent catchment functioning similar to *Andrews et al*. [2011]. We used the modified Kling‐Gupta efficiency (KGE) criterion to formulate calibration objectives simultaneously applied to time series of discharge (KGE_Q) and δ^2^H signature in streamwater (KGE_D) [*Kling et al*., 2012]. The KGE is a three‐dimensional and improved representation (Euclidean distance) of the widely used Nash‐Sutcliffe (NSE) criterion [*Schaefli and Gupta*, 2007]. The KGE ranges from infinity to a perfect fit of 1. Additionally, available data such as soil water and groundwater stable isotope signatures and soil moisture time series were used as qualitative “soft” data to aid model evaluation.

Modeling was started from 1 October 2003 with calibration from 1 June 2011. The first 4 years of precipitation data were used as a model warm‐up, principally to fill up storages, to stabilize storage concentrations, and to balance out any initiation effects on the water age calculations. Observed soil and groundwater isotope signatures were used to condition the initial composition of stores in the model. The hydrological and tracer characteristics of the calibration and test periods were broadly similar, though the former was slightly warmer and wetter (Table 2).

**Table 2:** Initial and Posterior Model Parameter Values and Ranges Expressed as Minimum and Maximum Values (in Parentheses) Derived From the Best Parameter (500 Parameter Sets) Population After Multiobjective Calibration^a^

	*a*	*b*	*r*	*K*	*α*	*upS_p_*	*satS_p_*	*lowS_p_*	KGE_Q	KGE_D	NSE_Q	R^2^_Q	NSE_D	R^2^_D
Initial range	[0.2, 0.8]	[0.0001, 0.1]	[0.2, 0.9]	[0.001, 0.1]	[0.1, 0.9]	[0, 1000]	[0, 1000]	[0, 1000]						
Unit	Day^−1^	Day^−1^	Day^−1^	Day^−1^		mm	mm	mm						
Calibration: 1 Jun 2011 to 30 Sep 2014	0.28 (0.2, 0.54)	0.0007 (0.0003, 0.002)	0.35 (0.25, 0.8)	0.02 (0.004, 0.09)	0.84 (0.51, 0.9)	804 (356, 980)	181 (148, 224)	550 (125, 990)	0.63 (0.55, 0.67)	0.59 (0.53, 0.62)	0.64	0.68	0.34	0.55
Test period: 1 Oct 2007 to 30 Sep 2009									0.62 (0.52, 0.66)	0.56 (0.5, 0.6)	0.6	0.62	0.26	0.41

a This indicates the accepted parameter variability and performance measures (KGE, best fit NSE, and R^2^) derived from the Pareto fronts in Figure 3 applied to the test period from 1 October 2007 to 30 September 2009. The initial parameter ranges were constrained based on previous tracer‐based studies [*Birkel et al*., 2010, 2015].

## 4 Results

### 4.1 Modeling Fluxes and Storage Dynamics

The calibrated model successfully captures the main streamflow variation with a KGE of 0.63 and a NSE of 0.64 (Figure 3 and Table 2). High flow periods, notably in summer 2011, December 2012 and 2013, and December 2014/January 2015 were effectively reproduced. Equally, low flows in March and September 2012, and June 2013 were simulated well. The model was less successful capturing rewetting phases; this is noticeable in the early summer of 2012, August 2013, and following the dry spring of 2014. In most of these cases, the model generally underpredicts the streamflow response. Despite this, the calibrated parameters transfer well to the test period of 2007–2011 with similar efficiency statistics, though again the model shows more limited skill in capturing rewetting (Table 2).

**Figure 3:**
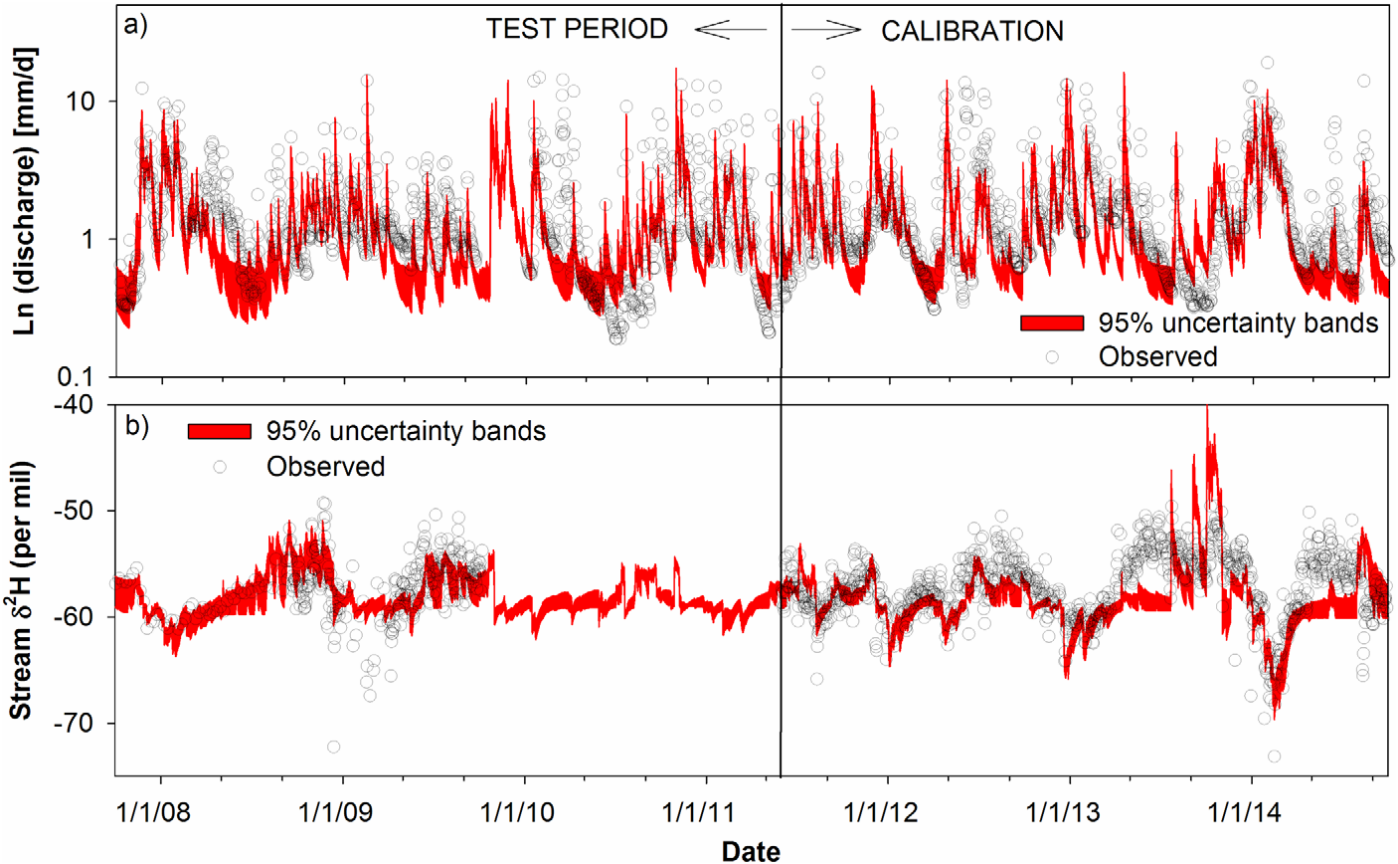
Final model structure calibrated against streamflow and deuterium in stream for the period from 1 June 2011 to 30 September 2014. This model was also used to simulate the test period from 1 October 2007 to 31 May 2011. Uncertainty bands based on 500 best parameter sets.

The main variations of δ^2^H in stream waters are also reproduced by the calibrated model with a KGE of 0.59, an R^2^ of 0.55, and a NSE of 0.34 (Figure 3 and Table 2). Seasonal dynamics, as stream composition shifts from more depleted winter values to more enriched summer signatures, are captured well. Also, periods of high fluxes are accurately simulated; particularly during wet winter periods, but also during the wet summers of 2011 and 2012. The calibrated mixing parameters for the main storage units decrease in the order *upSp* > *lowSp* > *satSp* as 804, 550, and 181 mm, respectively (Table 2). The main weakness of the isotope model is the limitation in accurately capturing the summer enrichment through evaporative fractionation despite its conceptualization. There is a clear underprediction of δ^2^H in the warmer drier summers of 2013 and 2014. This is compounded when the hydrological component of the models fails to capture the catchment rewetting, though δ^2^H overpredictions are evident in some events during the extreme summer drought of 2013 as the model simulated displacement of fractionated water from the saturation zone. Nevertheless, the calibrated parameters again transfer quite well to simulate the 2007–2009 isotope time series. There was only a small deterioration in model efficiencies, mainly explained by a larger snowmelt event in February 2009 which had unusually fractionated meltwaters which are not captured in the isotope simulations [*Birkel et al*., 2011a].

**Figure 4:**
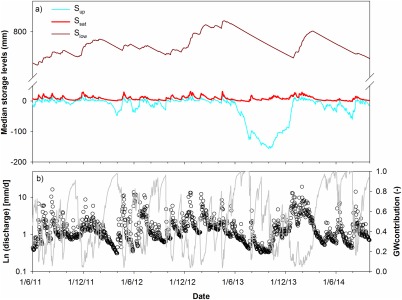
(a) Storage dynamics in the hillslope (S_up_), saturation area (S_sat_), and groundwater (S_low_) conceptual stores and (b) groundwater contributions (grey line) to streamflow (open circles). Modeled outputs are the medians of the 500 retained parameter sets.

The dynamic storage changes produced by the model while simulating the flow and tracer response can be disaggregated according to runoff sources (Figure 4a). The model structure dictates that the main variability in the runoff response to precipitation inputs is driven by the storage‐dependent connectivity between the upper hillslope (*S_up_*) and the saturation zone (*S_sat_*). In large precipitation events, positive storage in the latter generates fluxes during higher streamflows which correspond to the maximum expansion of the saturation zone. In smaller events, runoff is generated from the saturation zone which has a more limited extent. In contrast, the large groundwater store (S_low_) responds much more gradually to recharge events and subsequent dry‐period discharge, when groundwater becomes the main source of runoff such as in the summers of 2013 and 2014 (Figure 4b). Stream water in the calibrated model is mainly derived from fluxes from the saturated area; this accounts for approximately 70% of annual runoff. About 10% of this is generated by hillslope fluxes into the saturation zone. The remaining 30% of annual runoff are generated by groundwater fluxes directly into the stream.

In wetter periods, there are no storage deficits in *S_up_* and positive storage in *S_sat_* which drains to the stream, precluding high positive storage values (Figure 4). Groundwater storage increases during these wetter periods as recharge from *S_up_* occurs. During drier periods deficits develop in *S_up_* due to evaporative losses exceeding precipitation, but these are fairly small (<50 mm) with similar decreases in groundwater storage as recharge stops. However, the effects of the summer 2013 drought are particularly striking in the plot of the storage dynamics (Figure 4). This is evident in terms of the disconnection of *S_up_* and its storage deficit (∼150 mm) that subsequently accumulates and is not replenished until the end of the year. It is also apparent in the prolonged, gradual drawdown of groundwater storage, although this probably partly reflects oversimplification in the model structure as some groundwater recharge in summer and early autumn was detected in some groundwater wells [*Geris et al*., 2015]. This simplification also likely helps explain the poor simulations of rewetting on flows and associated errors in the modeled stream isotope response.

**Figure 5:**
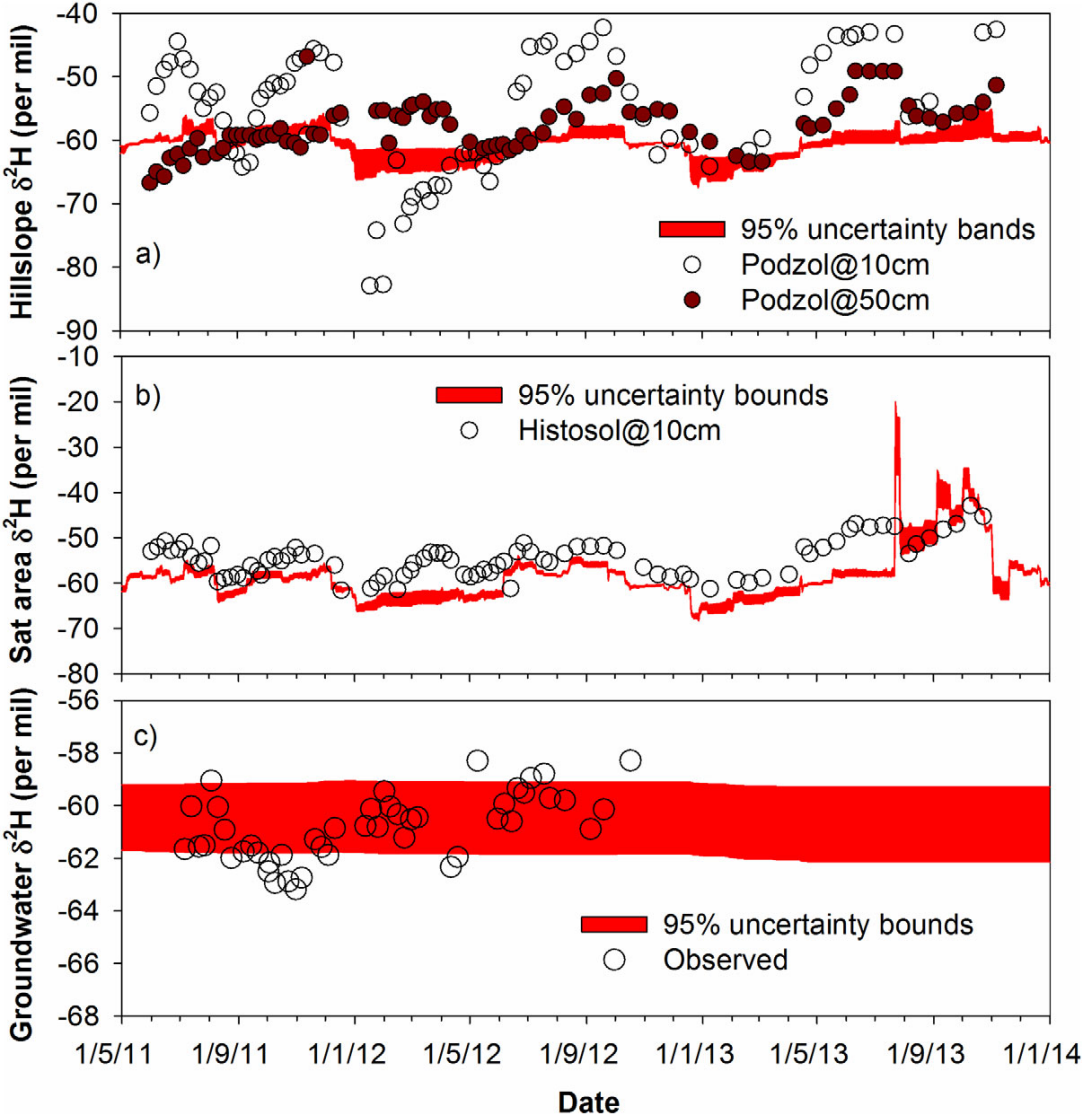
Final model structure calibrated against streamflow and deuterium in stream for the period from 1 June 2011 to 30 September 2014 used to simulate measured internal isotope states for the model stored compared to measured values in (a) the podzolic soils, (b) the riparian peats, and (c) groundwater. Uncertainty bounds based on 500 retained parameter sets.

A qualitative check on the internal isotope states produced in the three model units was available from measured isotope values (Figure 5). The isotope data were collected in the hillslope podzols in the upper (0.1 m depth) and lower (0.5 m depth) profile, and at 0.1 m depth in the riparian peat soils (Histosols) for 2 years. In addition, two deep groundwater wells (draining drifts >5 m deep) were sampled for 1 year. Although these point measurements are not strictly comparable (especially the two depths in the podzol), they give an indication of how realistic the internal states of the model are in terms of the mixing volumes which damp the isotope inputs in precipitation. Within the podzol at the hillslope (Figure 5a), the general direction of changes in the measured isotope dynamics is captured, but the model has much more restricted variation which can be perhaps viewed as an integration of the shallow and deeper soil waters, as well as that the model's lack of skill in capturing the effects of evaporative fractionation. Within the saturation zone (Figure 5b), the model is again effective in capturing the major direction of change at times of the major fluxes. However, the model does not reproduce the general enrichment of evaporative fractionation, especially during the summer. Nevertheless, the spike in simulated δ^2^H in the saturated area in summer 2013 shows the effect of this evaporative fractionation algorithm, albeit with an apparent overestimation of fluxes from the saturated area in a summer event (cf. Figure 3). The modeled groundwater composition (Figure 5c) is remarkably well mixed and constant, but in general brackets most of the measured values. Moreover, more recent surveys of 18 groundwater springs and seeps in the catchment revealed a mean δ^2^H composition of −61.5‰ with a standard deviation of 1.3‰, corroborating the representation of a generally well‐mixed groundwater source (C. Soulsby, unpublished data, 2015).

### 4.2 Ages of Stream Water and Fluxes From Different Landscape Units

**Figure 6:**
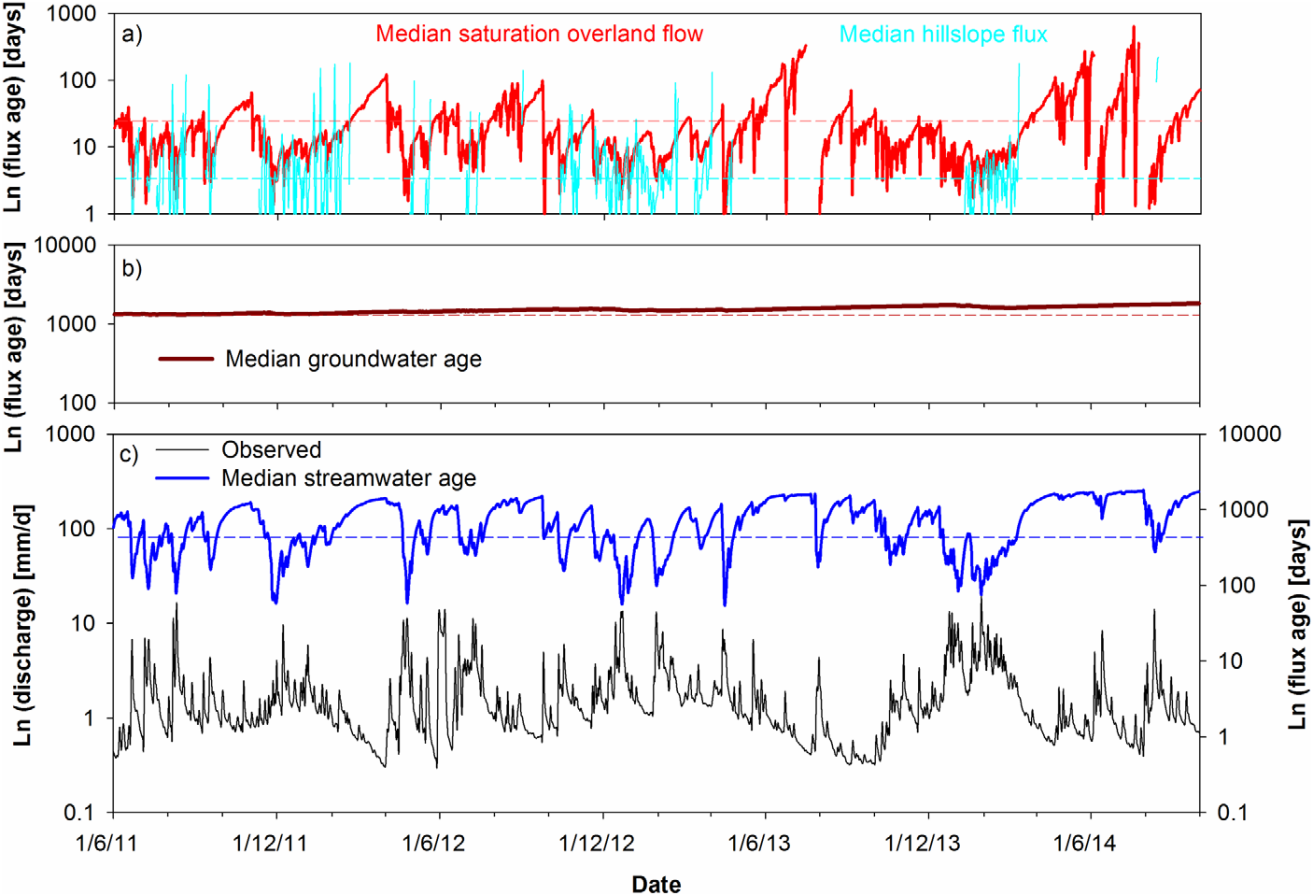
Median water flux age time series: (a) hillslope and saturation area flux age, (b) groundwater flux age, and (c) the streamwater age against discharge are shown over the calibration period from 1 June 2011 to 30 September 2014. The plots show the median of the 500 best parameter sets and the dashed lines indicate the MTT.

**Figure 7:**
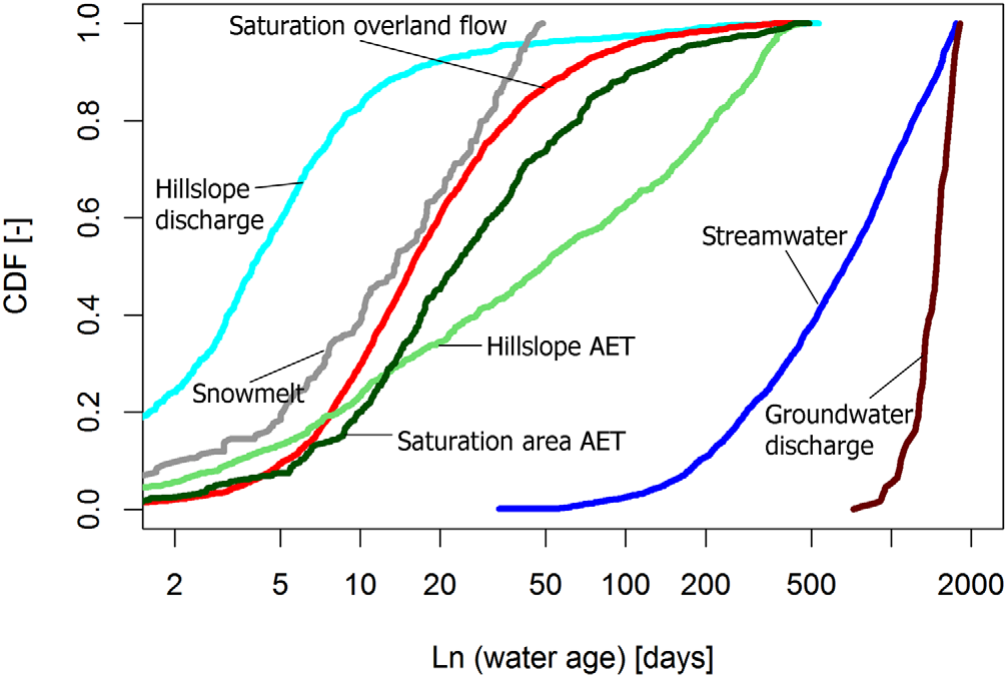
CDFs of water ages derived from the previous time series of water flux ages over the calibration period AET is actual evapotranspiration. Median CDFs of the 500 retained parameter sets are shown.

The fluxes modeled from the dynamic storage changes in the different landscape units allows the nonstationarity in water ages in fluxes to be tracked. Though there was considerable uncertainty in the estimates derived from the water ages from the 500 best parameter sets (see standard deviations in Table 3), the median of these retained model results give a constraint on the likely flux ages (Figure 6). The stream water age over the calibration period has an average transit time of 1.8 (±1.3 SD) years, but varied between daily averages of ∼4 years at low flows and ∼30 days at high flows (Figure 6 and Table 3). This was similar to the test period when 1.6 (±0.8 SD) years was the estimated mean transit time with a comparable daily age range. This average stream water age reflects the integration of the nonstationary water age distributions in the fluxes from the different landscape units which are visualized as time series in Figure 6 and shown as cumulative distribution functions in Figure 7.

**Table 3:** Water Flux Age and Storage Statistics (Mean and Standard Deviation in Parentheses) Derived From the 500 Retained Parameter Sets

Period	Q_up_	Q_low_	Q_sat_	Q	Hillslope AET	Sat Area AET	S_up_	S_low_	S_sat_
Unit	Day	Day	Day	Day	Day	Day	mm	mm	mm
Calibration	12 (31)	1426 (126)	29 (43)	603 (486)	109 (122)	50 (101)	−20 (41)	730 (34)	7.6 (5.7)
Test period	10 (35)	1333 (185)	30 (42)	583 (287)	86 (75)	53 (89)	−10 (25)	680 (64)	7.7 (5.1)
Wet	8 (26)	1266 (215)	9 (6)	308 (248)	15 (19)	26 (52)			
Dry	14 (46)	1314 (214)	54 (58)	983 (396)	120 (112)	61 (93)			

**Figure 8:**
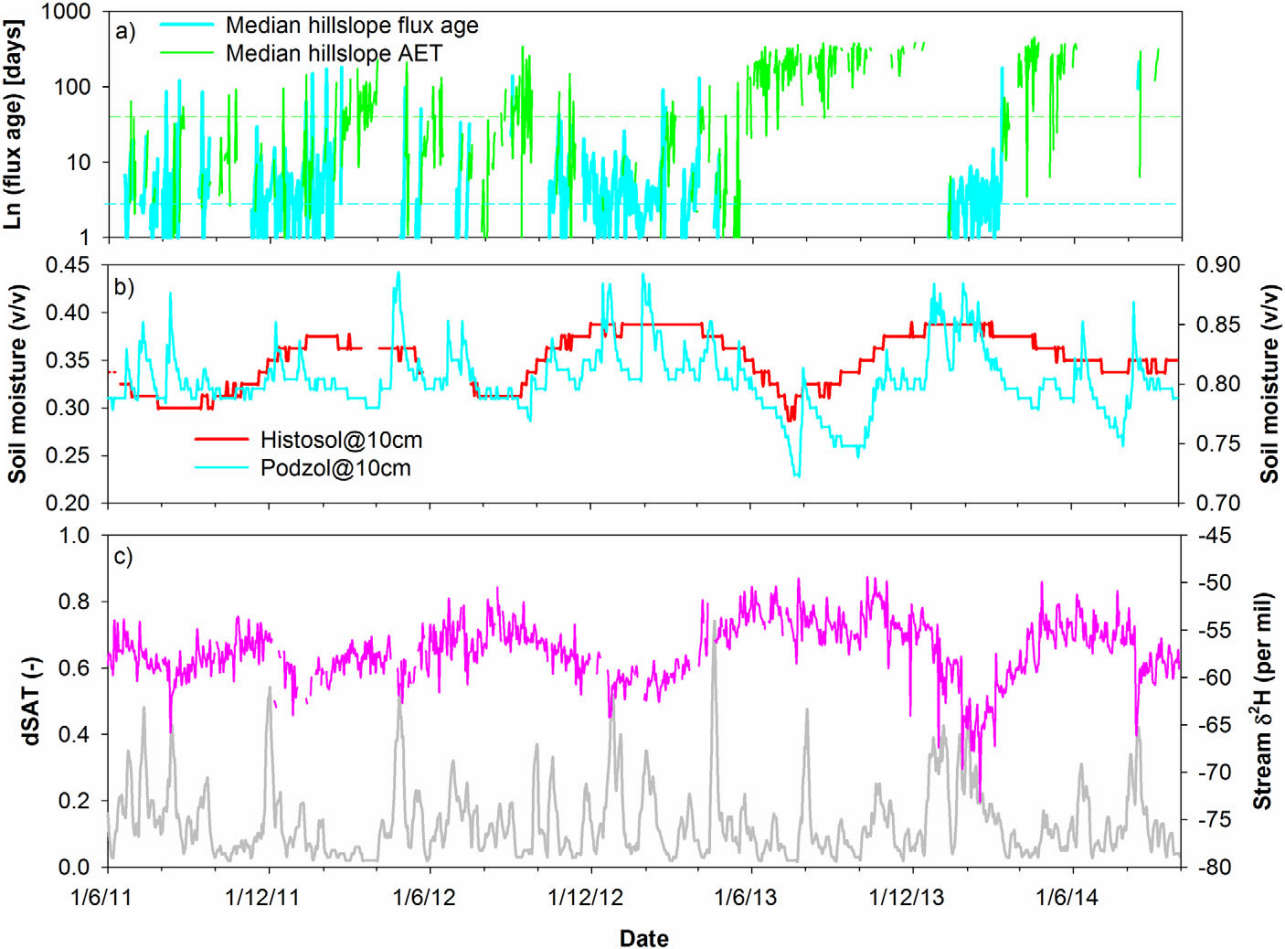
(a) Intermittent hillslope flux age and hillslope AET are shown in contrast to the catchment wetness state in form of (b) measured soil moisture and (c) modeled saturation area extent (dSAT) (grey line) against the observed isotope response in stream (pink line).

The nonlinear interactions between the different landscape units under different storage dynamics regulate the derived stream water age on any given day (Figure 6). These interactions are driven hydroclimatically depending upon precipitation event characteristics and antecedent conditions. This nonlinear connectivity control is because stream water is essentially a varying mixture of groundwater and overland flow from the riparian saturation area (mean ages of ∼4 years and 1 month, respectively), which may or may not be influenced by hillslope drainage (mean age ∼1 week) depending upon antecedent and event conditions. The age of saturation overland flow can vary between a few days to over 200 days. In the former case, youngest waters are transmitted to streams during more intense summer rainfall events with dry antecedent conditions. These situations result in no hillslope fluxes providing limited opportunities for riparian mixing. In contrast, during wetter periods, when there are hillslope fluxes into the saturation zone (Figure 6a) as well as larger volumes of in situ storage, there is greater mixing, increasing overland flow ages toward 10 days. These predicted periods of connection correspond closely to times when soil moisture, particularly in the hillslopes, is highest and the saturation area is most extensive indicating that the model is capturing these dynamics well (Figure 8). These connections also most likely occur in winter when isotope values are low. However, these highly connected periods are intermittent lasting for <20% of total time and rarely continue for more than a few months. Most of the time, the model predicts that the hillslope units are in storage deficit and disconnected from the saturation zone and stream channel, which is consistent with periods of lower soil moisture content and lower saturation area coverage. Such disconnection extended for up to 6 months in summer 2013.

As the catchment drains in drier periods, the estimated age of overland flow water reaching the stream increases toward 200 days and hillslope fluxes cease; eventually in prolonged dry periods overland flow also briefly ceases (Figure 6 and 8). Groundwater flux age is relatively constant at around 1400 days, though the model projects slightly increased age over the course of the study, largely as a result of the dry summers of 2013 and 2014 which, despite the wet period in December 2013/January 2014, resulted in limited simulated recharge. This is likely to be an artifact of the model where the simple structure does not accommodate the heterogeneity that allows some recharge to occur which would keep the ages lower. In wetter periods though, when high recharge through the hillslopes is modeled, the predicted groundwater age becomes younger (Figure 7).

The model output also allows for an estimation of the age of evaporated water fluxes from the wetland and hillslope, which are shown as CDFs in Figure 7. In the saturation area, the mean age for evaporated water is 2 months, but on the hillslope, this is generally about 3 months. The generally younger water evaporated from the saturation area reflects the wet conditions throughout much of the year and the lack of significant storage deficits. On the hillslopes, the drier periods where the hillslopes disconnect result in an increasing in the age of stored water and contributes to the comparatively older evaporated water.

### 4.3 Time Variance of the Interaction of Stream Water Age Distributions and Simulated Storage

**Figure 9:**
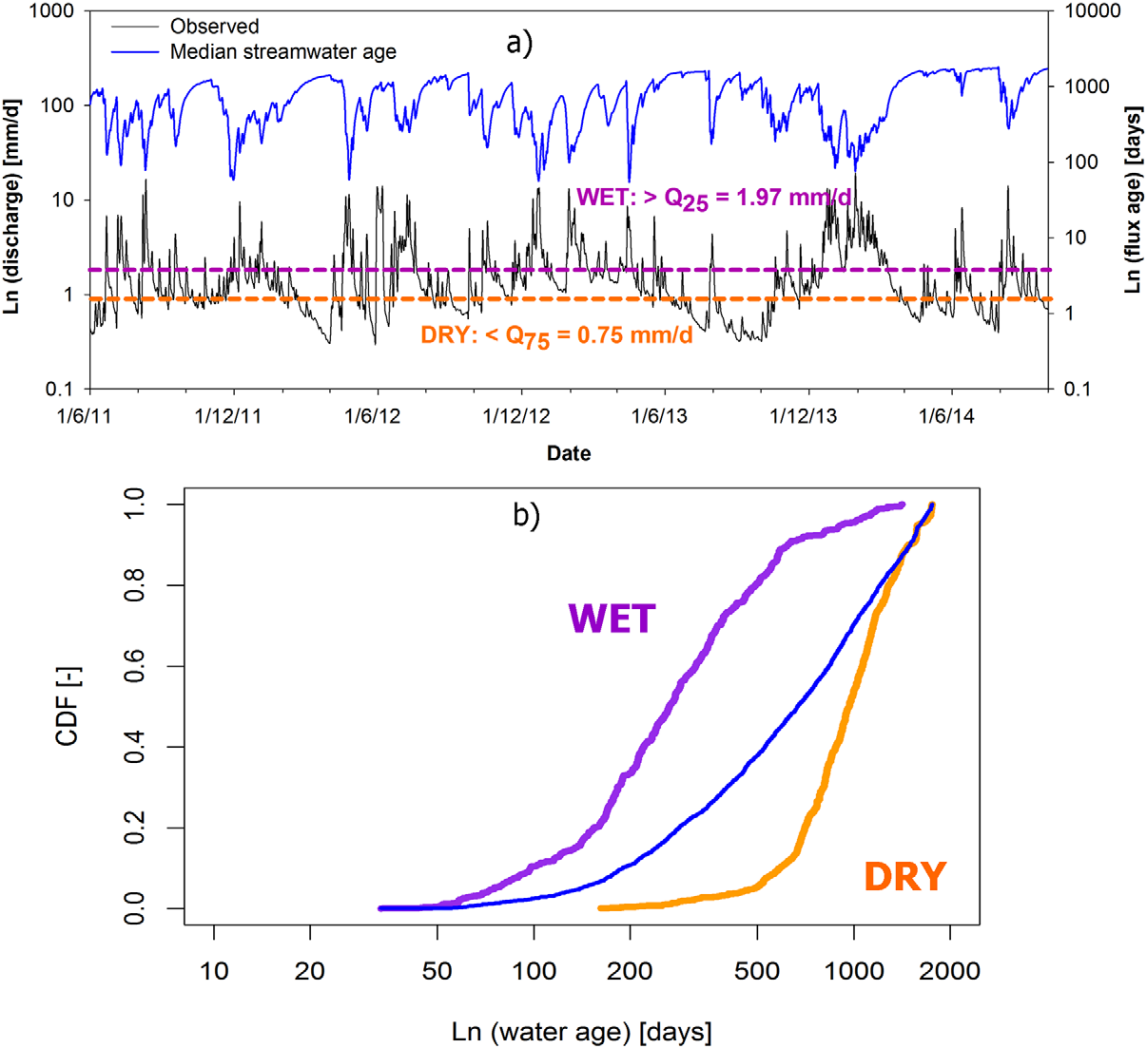
(a) The streamflow percentiles Q_25_ and Q_75_ were used to visualize the impact of (b) wet and dry (respectively) catchment state on streamwater age (the thinner blue line is the median of the long‐term data set identical to Figure 7).

To visualize the basic differences of system states in wet and dry periods and demonstrate more extreme conditions, the CDF of stream water ages (medians of the best 500 simulations) is summarized in terms of the upper and lower quartiles of the flow distribution in Figure 9. In wetter conditions at times of high precipitation, and flows >Q_25_, the mean stream water age is about 10 months. This coincides with periods of high connectivity and high turnover of stored water, which is most common during the winter time when evaporation is also relatively low. The saturation area extent is also greatest at these times (Figure 8). In drier conditions, at flows <Q_75_, older waters dominate with a mean age of about 2.5 years, reflecting the dominant groundwater source and smaller events tend to be generated almost entirely from the saturation area which serves to dilute the groundwater. These periods coincide with the lowest soil moisture contents when the hillslopes are disconnected.

**Figure 10:**
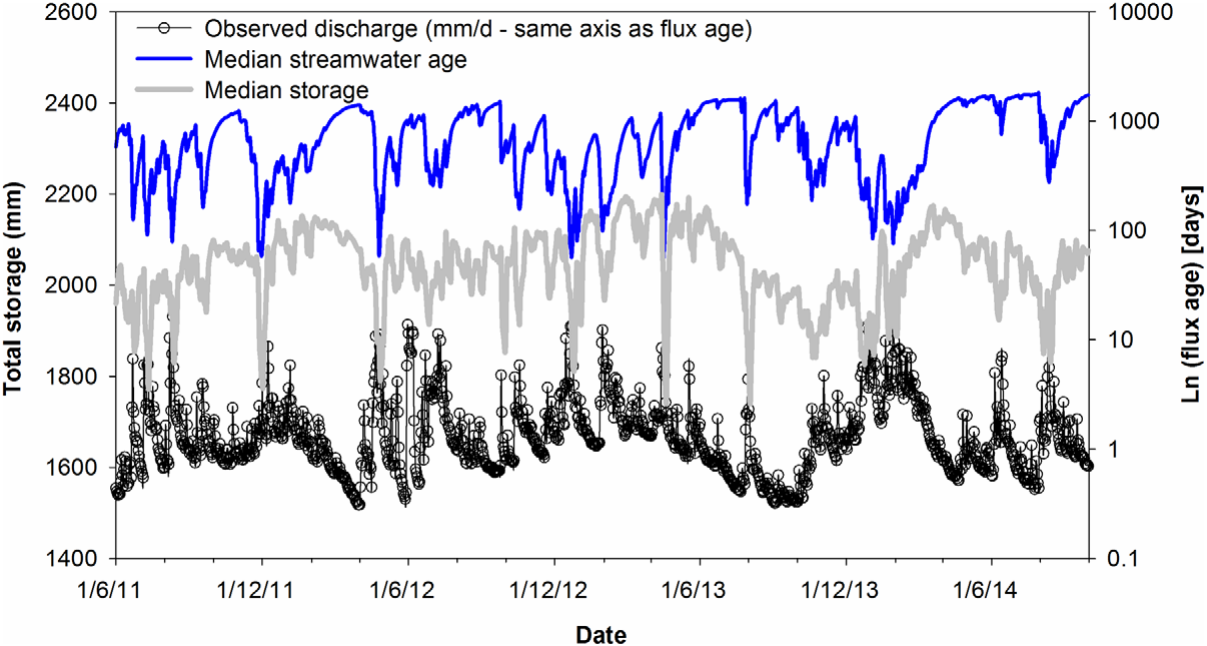
Modeled change in total effective storage involved in tracer mixing over the calibration period (median of 500 retained parameter sets) relative to flow and stream water age.

The effective volume of total storage involved in damping the tracers during transport derived from the model at these times show that the average storage at flows <Q_75_ is 1989 mm (±122 mm SD). For catchment states of >Q_25_, the mean storage is 1941 mm (±116 mm SD). This apparent decline in effective storage at the higher flows is also evident in the time series (Figure 10), particularly at the highest flows. Although seemingly counter intuitive, this reflects the effect of the larger extent of the saturation area and the lower mixing volumes which are activated compared to the larger hillslope storages which are reduced in spatial extent. This results in a greater proportion of precipitation being routed laterally to the stream channel from a larger area of the catchment, reducing groundwater recharge. In reality, of course, the total catchment storage is higher in these wetter periods, but the model indicates that the storage available to mix with incoming tracers is reduced. Previous work has shown that a simple storage‐discharge relationship (in the sense of *Kirchner* [2009]) is inadequate for modeling flows in this catchment, largely as a result of the effect of the saturation area and the resulting bypassing flows [*Birkel et al*., 2011b]. This is consistent with the process representation in the model here which achieves the tracer damping by conceptualizing and activating the dynamic mixing and flux from the saturation zone. This reduces the effective storage involved in the mixing due to the smaller, but more responsive storage compared to groundwater and the hillslope units.

## 5 Discussion

### 5.1 Simulating Flow, Storage, and Tracer Dynamics Using a Low Parameter Conceptual Model

The Bruntland Burn has a unique, rich isotope data set for use in tracer‐aided modeling. In this case, having almost 6 years of daily rainfall and streamflow isotope data. In this paper, this allowed us to make the significant scientific advance of being able to model and track water and tracer fluxes at a daily temporal resolution over a prolonged period with some extreme hydroclimatic periods (www.ceh.ac.uk/data/nrfa/nhmp/monthly_hs.html). The period included the wettest summer for 5 years in 2012, the coldest spring in 50 years and the warmest, driest summer for 10 years in 2013, and the wettest December/January for 11 years in 2013/2014. It also gave a sufficiently long time series for independent calibration and satisfactory testing of the model structure and parameter sets (Figure 3).

For catchments, where runoff generation processes are highly dynamic and involve a significant role for overland flow, capturing the nonlinear interactions between spatially distributed processes is critical for the coupled understanding and modeling of water and tracer transport processes [e.g., *Beven and Freer*, 2001]. In the case of the Bruntland Burn, the connectivity between steeper, freely draining hillslopes and the riparian saturation zone is fundamentally important to modeling both hydrological and isotope dynamics. However, in addition to these more rapidly responding units, a more gradually changing groundwater flux into the stream accounts for around 30% of streamflow and dominates flow for much of the year. Although the model structure was simplistic, and basic in terms of spatial distribution it was in keeping with the parsimonious philosophical basis that we sought and gave a reasonable representation of the system [cf. *Kirchner*, 2006; *Rinaldo et al*., 2006a, 2006b]. Moreover, the approach is potentially applicable in other catchments where peaty soils are important in generating dynamic saturation overland flow contributions [*Tetzlaff et al*., 2015].

### 5.2 How Do the Ages of Water Fluxes From Different Conceptual Stores Integrate at the Catchment Scale?

Recent work has demonstrated the importance of characterizing time‐variant water age distributions in hydrological fluxes and derivation of statistics such as the catchment mean transit time [*Rinaldo et al*., 2011]. Studies that have done this have often shown nonsmooth water age distributions in streamflow [e.g., *Botter et al*., 2010; *van der Velde et al*., 2010, 2012], which can reflect the time variance and nonlinearities in how different runoff sources are connected and their relative contribution to runoff generation [*Dunn et al*., 2010; *Birkel et al*., 2012]. In the Bruntland Burn, the stream water age at the catchment outfall can be viewed as the time‐varying integration of spatially distributed water fluxes from the main landscape units which each have their own age distributions (Figure 6). Storage dynamics within the landscape units drive the nonlinear connectivity between the hillslopes and riparian wetlands which in turn controls the streams hydrological response. The exact nature of this connectivity is modulated by antecedent conditions and event characteristics. These interactions then determine the “active” and “contributing” zones within the catchment, the relative proportion of water and tracer fluxes from the different landscape units and their respective ages which integrate to stream water age at the catchment outfall [cf. *Ambroise*, 2004]. These time‐varying spatial interactions are important drivers of water and tracer transport which are fundamentally important to process understanding and may not always be captured in purely lumped modeling approaches [e.g., *Hrachowitz et al*., 2013; *Soulsby et al*., 2015].

The model conceptualization effectively gives a dynamic mixing volume at each time step as fluxes from different landscape units in different wetness states are integrated. The resulting mean stream water ages (1.8 ± 1.2 years for the 500 best parameter sets for the calibrated model) are generally close and in the uncertainty range of previous estimates in the study catchment, such as the 1.9 (±0.9) years produced by lumped parameter models [*Hrachowitz et al*., 2010] or 1.3 (±0.3) years estimated by similar conceptual modeling frameworks though limited to only weekly data [*Birkel et al*., 2011b]. Indeed, a gamma model inversely fitted to the calibration time series used here also yielded a good fit (KGE = 0.58) with a mean transit time of 1.2 years, with an alpha parameter of 0.34 and a well‐constrained beta parameter. This is consistent with the ability of such lumped models to capture the integrated effects of the more and less responsive flow paths that are explicitly incorporated into the conceptual model [*Kirchner et al*., 2001]. The important role of the saturated riparian peats in generating the storm runoff response and providing a mixing volume for isotope inputs, together with the wet, low energy climate, probably explains the relatively stable nature of the stream water age distribution and average age over longer periods of record [*Tetzlaff et al*., 2014]. These mean stream water ages for the Bruntland Burn are intermediate compared to those reported across the Scottish Highlands, which can range from a few months in catchments with thin soils and impermeable geology in the much wetter west to over 4 years in catchments with higher storage in drift or fractured bedrock [*Hrachowitz et al*., 2009]. They are also comparable to recent estimates from the Plynlimon catchment in Wales; *Benettin et al*. [2015] showed that stream water age at Plynlimon, a similar upland, wet catchment in Wales, averaged around 1.5 years and comprised a time‐varying mixture of younger soil water (∼3 months old) and deeper groundwater (∼2 years old). Interestingly, they also found similar stream water age distributions derived from lumped and dynamic modeling approaches and a similar volume of storage (>2000 mm) effectively involved in tracer damping.

### 5.3 How Does Integration Vary Under Different Hydroclimatic Conditions and How Important are Nonlinearities in Hydrological Connectivity?

The nonstationarity in stream water age distributions could be clearly illustrated for the upper and lower quartiles of the flow distributions (Figure 9). In the wet scenario, the hillslopes and riparian wetland have strong connectivity with high proportions of relatively young water (<2 months) reaching the channel to maintain a stream water age that is usually <1 year. However, the persistent influence of older groundwater is still strong in mixing with these younger waters (Table 3). During such periods, hillslope storage and groundwater recharge can be reduced compared to drier conditions. This is because lower effective storage volumes are calculated by the model, which are dominated by the smaller (∼200 mm), but more dynamic (in terms of spatial expansion) stores in the riparian saturation zone as it covers a greater part of the catchment. This is consistent with recent findings of *Birkel et al*. [2015] and the “inverse storage effect” recently identified by *Harman* [2015]. In dry conditions, when the active zone is smaller, the groundwater store (>1000 mm) is the dominant source of streamflow with stream water ages >2 years, and the old water component accentuated due to drainage from the saturation zone being aging during the recession (Figure 6). However, for storm events with such dry antecedent conditions, individual events peaks are restricted to overland flow generated from within the saturation zone. Because of the limited storage at these times, the low mixing volumes result in very young (∼1 day) fluxes which can bring the stream water ages below 6 months. Other studies have found similar differences of wet and dry antecedent conditions [*Heidbuechel et al*., 2013; *Hrachowitz et al*., 2013; *Benettin et al*., 2015]. However, here with the unique daily data set, we can constrain some of the effects of nonlinear interactions between different landscape units in a more spatially explicit way.

### 5.4 Challenges and Prospects for Conceptual Models in Transit Time Modeling

The modeling approach used has the advantage of remaining relatively simple, but spatially explicit. It is, thus, a useful tool for applying in data rich catchments where dynamic saturation areas are important runoff sources as a framework for learning about catchment function and for hypothesis testing. In particular, the approach provides insight into spatial variability in storage dynamics, hydrological connectivity, and associated mixing processes that influence the age distribution of different source waters and ultimately stream water. The findings and approach are also particularly relevant to a wide range of northern temperate and boreal watersheds where dynamic activation of flow paths in peat is an important influence on the hydrological response [*Tetzlaff et al*., 2015]. Many such environments are experiencing rapid rates of environmental change, and models that have robust representation of catchment storages and flux ages are likely to be useful for predictive purposes [*Capell et al*., 2014].

However, the model can be improved to move beyond its current simplistic semidistributed partitioning of landscape units. In particular, empirical data from the site suggests that improved spatial resolution of the landscape features could improve the hydrological component of the model, particularly in terms of capturing the catchment rewetting after dry periods. Key areas of potential include the influences of microtopography in the valley bottom to distinguish connected and disconnected areas at the small scale [e.g., *Frei et al*., 2010]. Recent use of LiDAR in the catchment has shown restricted, more disconnected areas of saturation that are not captured by the simple lumped saturation algorithm used in the model and these are likely to contribute flows in the early rewetting phase as they connect and contribute to streamflow generation. In addition, there is evidence that aspect and land cover has an influence on evaporative losses and soil moisture deficits on the steeper catchment hillslopes with associated implications for groundwater recharge and slope‐wetland connectivity following drier periods [*Geris et al*., 2015]. Incorporating such processes would likely lead to improvements in flux estimates for the runoff model that will have indirect benefits for tracer modeling.

More direct issues to be addressed for improved tracer modeling revolve around the nonconservative behavior of isotopes. Of particular significance are the influences of evaporative fractionation in summer and fractionation in snowmelt. In the case of the former, there is evidence that the effects of summer fractionation are quite localized in quasi‐permanently connected pools and water tracks in the valley bottom saturation zone [*Blumstock et al*., 2015]. Thus, such model improvements could be linked to better representation of the valley bottom microtopography suggested above. For improved snowmelt characterization, this would require more systematic measurements of snow accumulation and melt [*Rodhe*, 1998]. Unfortunately, this is difficult, as snow pack accumulation is unpredictable and tends to be transient with rapid melt. Moreover, in most cases (i.e., rapid melt within a few days), marked fractionation of melt is not evident in stream water. However, even with improved snowmelt data, and improved spatially distributed conceptualization of evaporative fractionation or indeed evaporation, improvements to the model will come at the expense of more parameters, increased model structural complexity, and likely further model uncertainty due to identifiability and equifinality issues [*Beven*, 2012]. The appropriate level of model complexity in terms of process representation and the trade‐off with reduced identifiability depends on the exact objectives of the modeling.

## 6 Conclusion

We used a tracer‐aided conceptual runoff model to track daily water and isotope fluxes through a 3.2 km^2^ upland catchment in the Scottish Highlands. This model conceptualized three main hydrological response units: a dynamically saturated riparian zone of peat soils, freely draining hillslopes, and groundwater in extensive glacial drifts. The flux tracking was simulated with reasonable success over a 40 month period, which included some wet and dry spells that had return periods of around 10 years. The results showed that the estimated average age of stream water (1.8 years) was controlled by nonlinear connectivity between landscape units which determined the relative fluxes of younger water from saturation overland flow (∼10 days) in riparian peatlands and older, deeper groundwaters in the drift (∼4 years). Stream waters were youngest (∼1 month) in larger events when the extent of riparian saturation covered >40% of the catchment and connected fluxes from steeper hillslopes. In drier conditions, the age of stream water approaches that of groundwater. Fluxes between the main landscape units were driven by changes in storage. Relatively small (∼100 mm) changes in dynamic storage accounted for much of the water balance variation that controlled runoff generation. However, much larger total storage volumes (>1500 mm) were needed to account for the mixing processes that damped isotope signals in precipitation. In wet periods, this mixing primarily occurred in riparian wetlands. In drier periods, the hillslopes and groundwater provided the major mixing zones. While both the water and tracer flux dynamics were captured reasonably well, the simple model structure used had limitations in terms of simulating the rewetting of the catchment following dry periods and the effects of evaporative fractionation in the riparian wetlands. More complex spatially distributed models may help to capture these processes.
